# The Effect of Virulence and Resistance Mechanisms on the Interactions between Parasitic Plants and Their Hosts

**DOI:** 10.3390/ijms21239013

**Published:** 2020-11-27

**Authors:** Luyang Hu, Jiansu Wang, Chong Yang, Faisal Islam, Harro J. Bouwmeester, Stéphane Muños, Weijun Zhou

**Affiliations:** 1 Institute of Crop Science and Zhejiang Key Lab of Crop Germplasm, Zhejiang University, Hangzhou 310058, China; hu.luyang@foxmail.com (L.H.); 21716129@zju.edu.cn (J.W.); faisalislam@zju.edu.cn (F.I.); 2 Bioengineering Research Laboratory, Institute of Bioengineering, Guangdong Academy of Sciences, Guangzhou 510316, China; parker815@163.com; 3 Swammerdam Institute for Life Sciences, University of Amsterdam, 1000 BE Amsterdam, The Netherlands; H.J.Bouwmeester@uva.nl; 4 LIPM, Université de Toulouse, INRAE, CNRS, 31326 Castanet-Tolosan, France; stephane.munos@inra.fr

**Keywords:** parasitic plant, host, virulence, race, resistance mechanism, pathogen effector, evolution, *NLR*, *Orobanche*, *Striga*, interaction model

## Abstract

Parasitic plants have a unique heterotrophic lifestyle based on the extraction of water and nutrients from host plants. Some parasitic plant species, particularly those of the family Orobanchaceae, attack crops and cause substantial yield losses. The breeding of resistant crop varieties is an inexpensive way to control parasitic weeds, but often does not provide a long-lasting solution because the parasites rapidly evolve to overcome resistance. Understanding mechanisms underlying naturally occurring parasitic plant resistance is of great interest and could help to develop methods to control parasitic plants. In this review, we describe the virulence mechanisms of parasitic plants and resistance mechanisms in their hosts, focusing on obligate root parasites of the genera *Orobanche* and *Striga*. We noticed that the resistance (R) genes in the host genome often encode proteins with nucleotide-binding and leucine-rich repeat domains (*NLR* proteins), hence we proposed a mechanism by which host plants use *NLR* proteins to activate downstream resistance gene expression. We speculated how parasitic plants and their hosts co-evolved and discussed what drives the evolution of virulence effectors in parasitic plants by considering concepts from similar studies of plant–microbe interaction. Most previous studies have focused on the host rather than the parasite, so we also provided an updated summary of genomic resources for parasitic plants and parasitic genes for further research to test our hypotheses. Finally, we discussed new approaches such as CRISPR/Cas9-mediated genome editing and RNAi silencing that can provide deeper insight into the intriguing life cycle of parasitic plants and could potentially contribute to the development of novel strategies for controlling parasitic weeds, thereby enhancing crop productivity and food security globally.

## 1. Introduction

**Figure 1:**
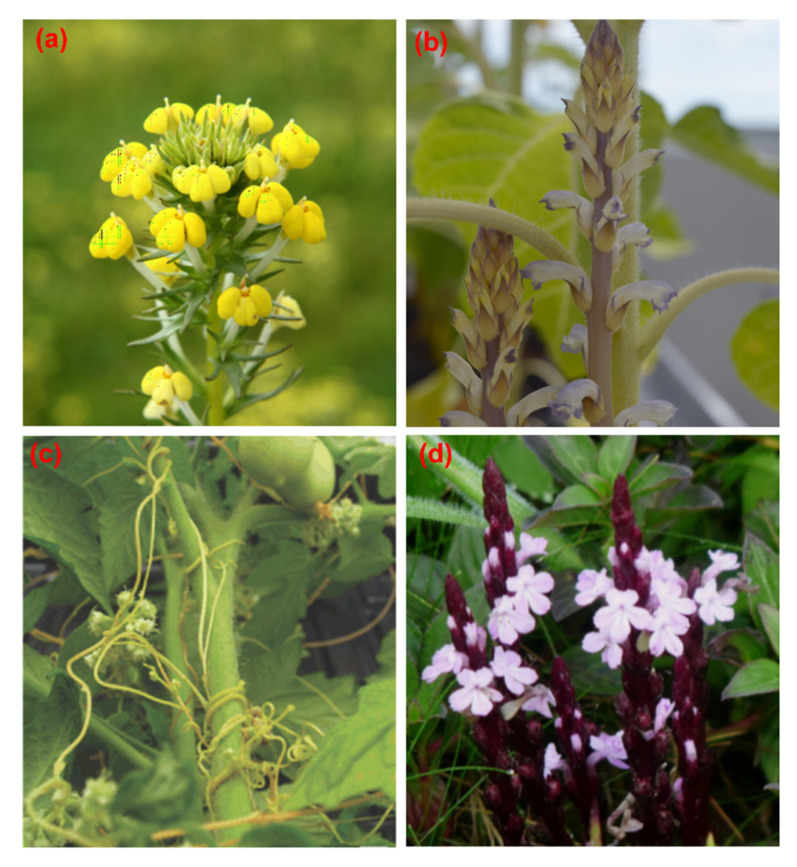
Some representative parasitic plant species. (**a**) *Triphysaria versicolor*, a hemiparasite, a photosynthetically competent species that, facultatively, parasitizes roots of neighboring plants; (**b**) *Orobanche cumana*, holoparasite, with absolute nutritional dependence on a host, mainly parasitizes roots of sunflower; (**c**) *Cuscuta pentagona*, holoparasite, also known as dodder, that parasitizes aboveground tissues of both monocot and dicot hosts; (**d**) *Striga gesnerioides*, an obligate hemiparasite that mainly parasitizes roots of cowpea.

Parasitic plants have a unique heterotrophic lifestyle in which they obtain water and nutrients from their hosts via an invasive root-like organ known as haustorium [1]. Parasitic plants occur in all terrestrial plant communities and ~4500 species have been described, distributed over 28 families, representing 1% of all dicotyledonous angiosperm species [2]. These parasites have independently evolved at least 12 or 13 times [3] and unprecedented horizontal gene transfer (HGT) [4] has contributed to their taxonomic and morphological diversity [1]. Some parasitic plant species attack crops and cause severe damage and yield losses, particularly in the Mediterranean, central and eastern Europe, Africa, and Asia [5,6]. Most research has focused on the genera *Orobanche, Striga, Cuscuta,* and *Viscum* (Figure 1). 

*Striga* and *Orobanche* species are especially difficult to control in the field due to their large seed banks and special parasitism traits [6], as well as the economic limitations in developing countries, where these parasites are most prevalent [3]. The life cycles of *Striga* and *Orobanche* species are similar because they coordinate with the life cycle of the host. The essential steps are germination, radicle growth to the host root, haustorium formation and attachment to the host root, establishment of a xylem–xylem connection, and the production of seeds [7,8]. The host–parasite interaction begins with the secretion of chemical signals by the host roots that induce the germination of parasite seeds and are called germination stimulants [9]. Accordingly, the inhibition of parasite seed germination is a primary target for parasitic weed control [10]. Almost all germination stimulants discovered thus far belong to the carotenoid-derived strigolactone (SL) family [11]. Recent studies have shown that the breeding of crops showing limited exudation of SLs from the root is an effective strategy to achieve resistance to *Orobanche* and *Striga* [12,13,14]. We, therefore, discussed low SL levels as a natural resistance mechanism in host plants as well as biotechnological strategies to induce this trait. Other practical methods to control parasitic plants have been extensively reviewed but are often unsuccessful in the long term because the parasite evolves faster than the resistant host, leading to the emergence of distinct races or pathotypes with renewed virulence [6,10,15,16,17,18]. 

The existence of host-specific races suggests that parasites have evolved complex mechanisms to overcome potential host resistance, but most reviews overlook this aspect and focus on the host’s resistance mechanisms. Here, we considered recent examples of virulence and race evolution in parasitic plants before looking at host resistance mechanisms in the context of canonical resistance genes (*R* genes) encoding proteins with nucleotide-binding and leucine-rich repeat (*LRR*) domains, often termed “*NLR* proteins”. We used these to develop a plausible model explaining the molecular basis of host–parasite interactions. We also summarized current genomic resources for parasitic plants and discussed the functions of known virulence genes and their roles in the evolution of host-specific races of parasitic plants. 

## 2. Virulence Evolution in the Family Orobanchaceae 

### 2.1. Definition of Race in Parasitic Plants

In biological taxonomy, race is an informal rank below the level of subspecies that may be defined according to any identifiable characteristic (e.g., chromosomal race, geographical race, or physiological race), but the differences are relative rather than absolute. When we talk about race in the context of parasitic plants it usually refers to physiological race, which means a group of individuals that do not necessarily differ in morphology from other members of the species but have distinct physiology or behavior. In parasitic plants, a race signifies a genotype that has the capacity to parasitize on a certain genotype of host plant. For example, *Orobanche cumana* (*O. cumana*) races are classified according to the resistance/susceptibility of a set of sunflower lines carrying different resistances’ genes. A new nomenclature, similar to the one used for downy mildew pathogens, was proposed [19]. A physiological race may be an ecotype (subgroup of a species that has adapted to a different local habitat), perhaps defined by a specific food source. Parasitic plant species tied to no geographic location often have races that are adapted to different hosts, but these are, so far, at least difficult to distinguish genetically. 

### 2.2. History of Race in Parasitic Plants

The family Orobanchaceae is part of the order Lamiales, which comprises annual herbs as well as perennial herbs and shrubs. With the exception of the nonparasitic genera *Lindenbergia*, *Rehmannia,* and *Triaenophora*, members of the Orobanchaceae parasitize the roots of other plants and display all known types of plant parasitism: facultative parasitism, obligate parasitism, hemiparasitism, and holoparasitism. *Striga* and *Orobanche* are widely studied because of their impact on agriculture. For example, *O. cumana* (sunflower broomrape) causes yield losses of up to 80% [20,21,22]. In sub-Saharan Africa, up to 60% of the arable land used to cultivate cereals and grain legumes is infested with one or more *Striga* species [23]. Within parasitic plant species, races can be distinguished. For example, seven races of *Striga gesnerioides* parasitizing cowpea (*Vigna unguiculata*) have been identified [24] and eight races of *O. cumana* parasitizing sunflower (*Helianthus annus*) [25].

The race evolution history of *O. cumana* has been studied on sunflower and wild species of the Asteraceae, mainly *Artemisia maritime* (sea wormwood) [26]. A mature *O. cumana* plant can produce 50,000–500,000 dust-like seeds, which remain viable in soil for up to a decade [15]. The long viability of *O. cumana* seeds limits sunflower production in contaminated fields, mainly in Eastern Europe and Asia, and is found in Spain, France, Turkey, Russia, Ukraine, Israel, Kazakhstan, and China. The virulence or pathogenicity of *O. cumana* has evolved rapidly with the increasing global production of sunflower, especially in Russia, eastern Europe, and Asia since the 1920s [19]. *O. cumana* races were first discussed by local sunflower breeders in Russia in 1920 [27]: race A in the Saratov and Voronezh regions did not attack local sunflower crops, whereas race B in the Rostov and Krasnodar regions was highly virulent against the same sunflower variety. Since then, *O. cumana* has evolved quickly from race A to race H and has parasitized local sunflower varieties for 100 years [19,27]. 

The dispersion of *O. cumana* races has been systematically reviewed [19]. However, there is no worldwide consensus on the sunflower lines used to identify *O. cumana* races because different countries and regions favor distinct sunflower lines or hybrids in local breeding practices for identification purposes. Therefore, it is difficult to compare results from experiments carried out in different parts of the world for the characterization of *O. cumana* race structure. Race identification is usually carried out by counting emerged shoots of *O. cumana* on distinct sunflower lines or hybrids.

**Figure 2:**
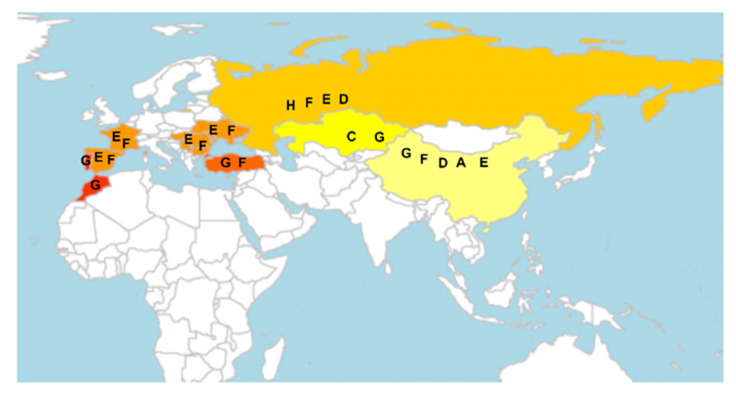
Current distribution and virulence level of *Orobanche cumana* in the world. Distribution range of *O. cumana* in the worldwide regions updated to December 2019. Although different levels of virulence are not comparable horizontally, it is notable that the parasitic plant *O. cumana* evolves fast with the increasing production of sunflower. Letters in the figure refer to distinct virulence levels. Race A: China; Race B: None; Race C: Kazakhstan; Race D: China and Russia; Race E: Bulgaria, China, France, Hungary, Moldova, Romania, Russia, Serbia, Spain, and Ukraine; Race F: Bulgaria, China, France, Hungary, Moldova, Romania, Russia, Spain, Turkey, and Ukraine; Race G: Bulgaria, China, Kazakhstan, Romania, Russia, Spain, Turkey, Ukraine, Morocco, and Portugal; and Race H: Russia.

Figure 2 represents the worldwide occurrence of race structure of *O. cumana*. The races E, F, and G are formerly the most commonly reported all over the world, but races F, G, and H are now the most prevalent in Spain and in several countries around the Black Sea, whereas races A, D, E, F, and G are the most prevalent in China [28,29]. A new race (G_KE_) responsible for ~80% yield losses was identified in Morocco in 2016 [30]. In 2017, race G was found for the first time in Portugal [31] (Figure 2). 

## 3. The Mechanisms of Virulence-Specific Resistance in Host Plants

### 3.1. Phenotypic Aspects 

The race or pathotype of parasitic plants is often determined by quantification of the infection level. The sunflower lines 2603 and P-96 are typically used as controls because the first is susceptible and the last is resistant to *O. cumana* race F [20]. Rhizotrons, pots, and field experiments were used to characterize all sunflower recombinant inbred lines for resistance to *O. cumana* race F at three life stages: (1) early attachment of the parasite to the sunflower roots, (2) young tubercle, and (3) shoot emergence [20]. This showed that the number of healthy tubercles at stage 3 is the trait best correlating with the number of emerged broomrape shoots in the field. Other researchers have counted the necrotic tubercles (post-haustorial/secondary resistances) in the resistant line or the number of successfully established radicles allowing the development of root tubercles on the susceptible line [32,33,34]. Another strategy to determine the successful infection by parasitic plants is to measure host plant parameters such as height, weight/biomass, photosynthesis, leaf CO_2_ assimilation rates, transpiration rate, stomatal conductance, vapor pressure deficit, and leaf carbon, nitrogen, potassium, phosphorus, and magnesium levels [35,36,37]. For example, the effects of *Striga* on susceptible rice (*Oryza sativa*) genotypes included 30–65% stunting of the main stem and the inhibition of photosynthesis and CO_2_ assimilation in 30-day-old plants (and even more profoundly in 45-day-old plants), whereas these effects were not evident in resistant genotypes, resulting in high grain yields in the field [35]. Interestingly, the comparison of susceptible and resistant sunflower varieties in response to *O. cumana* revealed no physiological differences between the infected and non-infected cohorts of the resistant cultivar (cv) after 23–51 days of planting, including photosynthesis, transpiration rate, stomatal conductance, vapor pressure deficit, nonphotochemical quenching, and chlorophyll levels [36]. However, significant differences were found in the levels of the macro-elements potassium, phosphorus, magnesium, and sulfur between the infected and non-infected plants during the early stage of parasite development. The mineral and carbon content were higher in the broomrape infected sunflower, as compared to the non-infected ones after 31 days of planting [35]. Sunflower leaf nitrogen content, however, was 42% lower in broomrape-infected plants after 56 days of planting, which can be explained by the reduction in mesophyll cells per area leaf and a delay in leaf senescence [36]. 

Resistance that occurs in multiple layers is often associated with the accumulation of compounds such as H_2_O_2_, peroxidases, β-glucanase, and callose in the case of *Orobanchecrenata* vs. pea (*Pisum sativum*), or 7-hydroxylated simple coumarins in the case of *Orobanche cernua* vs. sunflower [38]. To reinforce the cell wall, resistant plants deposit lignin in the endodermis and pericycle cells at the penetration site, as seen in the cases of *O. crenata* vs. vetch (*Vicia* spp.), faba bean (*Vicia faba*), pea, chickpea (*Cicer arietinum*) and lentil (*Lens esculenta*), *O. cumana* vs. sunflower, and *Striga hermonthica* vs. rice [39,40,41]. Genetic analysis has also confirmed that lignification and secondary wall formation promote resistance during incompatible interactions between cowpea and *S. gesnerioides* [42] and between rice and *S. hermonthica* [41].

### 3.2. Histological Aspects 

**Figure 3:**
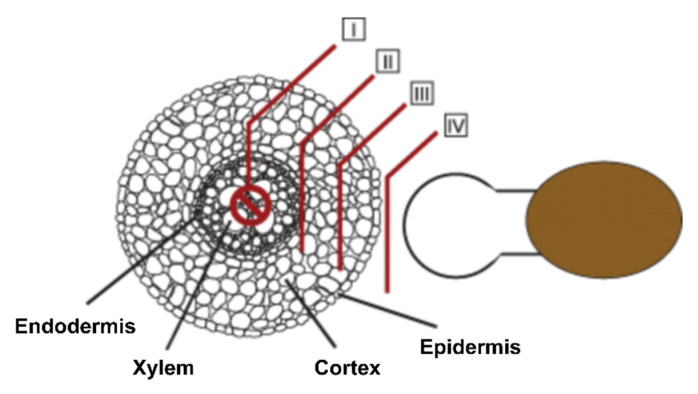
Schematic illustration of the different root cell layers where resistances cans occur. Four layers of incompatibility described in the text are presented. (**I**) Incompatibility expressed after vascular connection, which was observed in *Arabidopsis*, cowpea, and in rice cultivar Nipponbare. (**II**) Endodermis blockage, which is observed in rice cultivar Nipponbare as well as in cultivar Koshihikari. (**III**) Mechanical barrier in the root cortex, observed in interaction with *Lotus japonicus* and occasionally with *Phtheirospermum japonicum*. (**IV**) Incompatibility preventing attachment, observed in interaction with *P. japonicum*. Parasite plant (right) and host plants (left, shown as a transverse section of a root). Adapted from Yoshida et al. [44].

**Figure 4:**
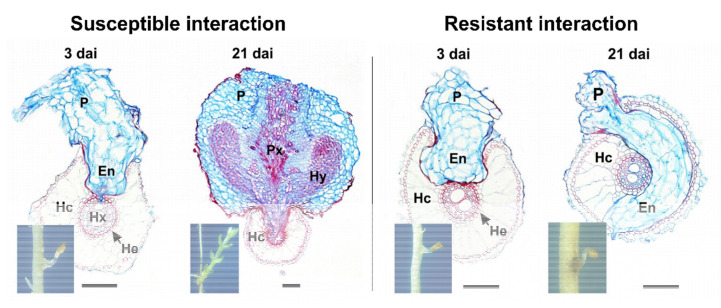
Host resistance to parasite establishment. Transverse sections of embedded tissue of susceptible (cv. Kasalath) and resistant (cv. Nipponbare) rice roots 3 and 21 days after inoculation with *Striga hermonthica*. In the susceptible interaction, the parasite penetrates the cortex and endodermis and connects to the xylem vessels of the host, allowing the haustorium to differentiate. In contrast, in the resistant interaction, although the parasite penetrates the cortex, it is unable to breach the endodermal barrier and grows around the host vascular cylinder. The parasite is unable to access host water and nutrients and the haustorium does not differentiate and the parasite dies. The scale bar represents 0.1 mm. En, endophyte (internal part of haustorium); Hc, host root cortex; He, host endodermis; Hx, host xylem; Hx–Px, host–parasite xylem continuity; Hy, hyaline body; P, parasite haustorium; and Px, parasite xylem vessels. Adapted from Gurney et al. [45].

To ward off infection by parasitic plants, host plants can deploy several defense mechanisms. The first line of defense is a physical barrier (the cuticle and cell wall) supported by the constitutive production of metabolites that deter the invader. Successful penetration of the root cell layers and the establishment of host–parasite vascular connections are necessary for *Orobanche* and *Striga* to survive [8]. The host can, therefore, block parasite development at the epidermis, in the cortex, at the endodermis, and inside the central cylinder [23,43,44] (Figure 3). In rice cultivar Nipponbare, which shows strong resistance to *S. hermonthica*, parasite development is inhibited at the cortex, suggesting that the host blocks signaling pathways required for the parasite to penetrate between endodermal cells [44,45] (Figure 4). In sunflower, the major resistance gene *HaOr7* prevents the connection of *O. cumana* to the sunflower root vascular system [46]

### 3.3. Chemical Aspects 

The life cycle of parasitic plants begins when seed germination is promoted by root exudates from a presumptive host. The inhibition of seed germination is, therefore, a key target for parasitic weed management [47] and this can be achieved by reducing the amount of SLs exuded by host roots [12]. Early studies identified host germplasm that produces lower levels of *Orobanche* germination stimulants in root exudates, yielding resistant varieties of pea, chickpea and *Lathyrus* spp. [32,48,49,50,51], faba bean [52], and sunflower [53,54,55]. Similarly, host germplasm that produces lower levels of *Striga* germination stimulants were identified in sorghum (*Sorghum bicolor*) [11] and maize (*Zea mays*) [56], and such traits have already been used to breed resistant sorghum varieties [57]. 

Initially, the nature of the stimulant was unclear. To collect sunflower root exudates, seedlings were transferred to sterile distilled water for 1–5 weeks after germination and cultivated for three days before preconditioned *O. cumana* seeds were incubated in the root exudate solution [54,55]. In these experiments, the germination of *O. cumana* seeds was stimulated using one ppm GR24, an artificial SL analog. The exudates of different sunflower genotypes had different effects on the broomrape seedlings, indicating that chemicals in the exudates have an effect on *O. cumana*. The development of high-performance liquid chromatography connected to tandem mass spectrometry allows the identification of specific compounds that act as germination stimulants, revealing that the abundance of SLs in pea root exudate correlates with resistance [14]. Most germination stimulants discovered thus far are SLs, and this suggests that resistance may involve the reduced secretion of SLs in root exudates [58]. 

SLs were first found in cotton (*Gossypium* spp.) root exudates as a potent germination stimulant of *Striga lutea* [59]. Subsequent research revealed that SLs stimulate hyphal branching of arbuscular mycorrhizal fungi [60,61], but also function as endogenous hormones to inhibit shoot branching or tillering [62,63]. SL biosynthesis begins with all-trans/9-cis β-carotene isomerase (*DWARF27* or *D27*), which converts all-trans-β-carotene to 9-cis-β-carotene [64,65]. In the next step, carotenoid cleavage dioxygenase 7 (*CCD7*) cleaves 9-cis-β-carotene into the volatile β-ionone and 9-cis-β-apo-10′-carotenal [64,66]. This cis-configured intermediate is the substrate for *CCD8*, which catalyzes a combination of reactions including repeated dioxygenation and intramolecular rearrangements to yield carlactone and the C_8_-product ω-OH-(4-CH_3_) heptanal [64,67]. 

Carlactone is the precursor of all SLs, although the later reaction steps are not completely understood [68,69]. In *Arabidopsis thaliana*, carlactone is converted into carlactonoic acid by the cytochrome P450 monooxygenase *MORE AXILLARY GROWTH 1 (MAX1*), followed by methylation by an unknown enzyme and hydroxylation by lateral branching oxidoreductase into a yet unidentified SL [70,71]. In rice, *MAX1* homologs convert carlactone into 4-deoxyorobanchol and orobanchol [72,73]. The biosynthesis and transport of SLs was reviewed in detail [69].

It is currently unclear whether the germination of parasitic plants involves additive, synergistic, and/or antagonistic effects in response to the usually multiple germination stimulants produced by their hosts. There is evidence that germination stimulants are involved in species-dependent and race-specific effects because the synthetic SL GR24 induces germination up to ~70–90% in *Orobanche ramosa*, *O. cumana*, and *O. minor*, but only up to ~50% in *S. hermonthica* [74,75]. In vitro bioassays with *O. minor* seeds revealed that the activity of 1 μM heliolactone is similar to that of GR24 after 24–72 h (75% germination) but only ~25% of *O. cumana* seeds germinated, suggesting additional compounds may be required for the latter species [76,77,78,79,80]. Moreover, additive or antagonistic effects were observed between two SLs [81]. For example, strigol and orobanchol together germinate 24% of *O. cumana* seeds, while orobanchol alone induces 64% germination of *O. cumana* [81].

More evidence of host-dependent parasite germination comes from the analysis of SL biosynthesis in different hosts. The first step, the formation of carlactone, is common to all hosts, but the next steps are species-dependent. In many species carlactone is converted to carlactonoic acid, for example, carlactonoic acid is converted into methyl carlactonoate and then converted into heliolactone in sunflower [72], while in rice carlactonoic acid is converted to 4-deoxyorobanchol [73]. Carlactonoic acid is the precursor for strigol in moonseed, for sorgomol in sorghum, and for strigol in cotton [72,82]. 

The analysis of SLs in autotrophic plants such as *Arabidopsis* has revealed that the receptor for endogenous strigolactones in nonparasitic plants is encoded by *DWARF 14 (D14)*, while the strigolactone receptor in root parasitic plants, responsible for the detection of host strigolactones, is encoded by *HYPOSENSITIVE TO LIGHT/KARRIKIN INSENSITIVE2 (HTL/KAI2)* [83,84,85,86]. Intriguingly, genes encoding strigolactone biosynthetic enzymes as well as the receptor D14 have also been identified in parasitic plants [87]. Several *HTL/KAI2* strigolactone receptors from parasitic plants have been functionally characterized [88,89,90,91,92]. For example, *ShHTL7* (*Striga hermonthica* HTL protein) is found to be a very sensitive SL receptor that binds with several natural strigolactones [88].

### 3.4. Genetic and Genomic Aspects 

#### 3.4.1. R Genes against *Orobanche cumana*

Genetic studies related to the virulence/race of *O. cumana* have been poorly described. However, an avirulence gene interacting with *Or5* resistance gene in sunflower (see below) was characterized [93] and was mapped [94]. The *O. cumana* genome sequence will help to identify the avirulence genes [95]. The sunflower genes *Or*1, *Or*2, *Or*3, *Or*4, *Or*5, and *Or*6 confer resistance to *O. cumana* races A, B, C, D, E, and F, respectively, and are inherited as single dominant alleles [96,97,98]. Resistance to race F has also been associated with two recessive alleles [91], two partially dominant alleles [99], and multiple quantitative trait loci (QTLs) [19]. Preliminary results suggest that resistance to race G may be conferred by a single dominant allele [100] or a single recessive allele [101]. However, all these studies used traditional crosses to determine whether the resistance gene is transmitted in a dominant or recessive manner, with no indication of the candidate genes or their potential functions. The sequencing of the sunflower genome should help to identify resistance genes [102].

The genomic location of an *O. cumana* resistance gene in sunflower was recently verified by bulk segregant analysis combined with genotyping-by-sequencing technology. Two major QTLs associated with resistance were resolved to sunflower chromosome 3 (*or3.1* and *or3.2*) and the former maps to the same region as *Or5* (conferring resistance to race E) whereas the latter is associated with markers of resistance to race G. Exploration of the first region (31.9–38.48 Mb) revealed 123 candidate genes, including a known disease resistance gene (*HanXRQChr03g0065841*) encoding an oxygen-dependent choline dehydrogenase and *FAD/NAD(P)*-binding domain [103]. The second region (97.13–100.85 Mb) contained 71 candidate genes, including one with an *NLR* domain (*HanXRQChr03g0076321*) that is often found in resistance gene (*R*) products [104], such as the *Arabidopsis* R proteins *RPM1* and *RPS5* [105]. The sunflower orthologs of *PRM1* and *PRS5* are both induced in the *O. cumana*-resistant cultivar JY207 following inoculation with the parasite, but there is no change (or even a slight fall) in the susceptible cultivar TK0409 compared to non-inoculated controls [40]. The *HaOr7* resistance gene to race F from Spain was identified by a map-based cloning approach and encodes a receptor-like kinase [46].

#### 3.4.2. R Genes against *Striga gesnerioides*

Another well-studied example of race evolution in parasitic plants is *S. gesnerioides* (Figure 1d), which has multiple races that significantly affect cowpea production in sub-Saharan Africa [106]. Crossing and backcrossing experiments among resistant and susceptible cowpea cultivars indicated a monogenic resistance locus with a dominant inheritance pattern [107,108,109]. This led to the proposed designations of *Rsg1*, *Rsg2,* and *Rsg3* for the genes present in cowpea cultivars B 301, IT82D 849, and SUVITA-2, respectively [18]. Later studies in cowpea identified amplified fragment length polymorphism markers tightly linked to different race-specific *S. gesnerioides* resistance genes [110] and the microsatellite/simple sequence repeat marker SSR-1 co-segregating with *S. gesnerioides* race 3 (SG3) resistance [111], which was ultimately identified in a cowpea gene-space sequence read [112]. The gene was named *RSG3-301* (resistance to *S. gesnerioides* race 3 in cowpea cultivar B301) and was shown to encode an R protein with an *NLR* domain [113]. When *RSG3-301* expression is knocked down by virus-induced gene silencing (VIGS) in the multirace-resistant cowpea cultivar B301, *S. gesnerioides* can invade the endodermis and establish xylem–xylem connections with the host vascular system [113], suggesting that *S. gesnerioides* may interfere with the regulation of *NLR* proteins to overcome host plant defenses. 

Recently, a transcriptome study focusing directly on parasitic plants rather than their hosts has revealed that candidate haustorium-specific genes in *T. versicolor* and *S. hermonthica* are significantly enriched for aspartyl protease, peroxidase, and *NLR* protein domains [114]. Moreover, a novel decoy effector *SHR4z* was recently identified from the haustorium of *S. gesnerioides* that can suppress the hypersensitive response in host cowpea plants to boost parasite growth. *SHR4z* has significant homology to the short *LRR* domain of somatic embryogenesis receptor kinase (*SERK*) proteins and functions by binding to *VuPOB1*, a positive regulator of the hypersensitive response [115]. 

#### 3.4.3. Virulence Genes in Parasitic Plants 

Parasitic plants are more complex organisms than microbes and pathogens. Parasitic plants could possess specific proteins involved in virulence and they must be considered as pests because they also induce diseases in a host plant [116]. Insight into the distinction between virulence genes (pathogen effectors) and host resistance genes could be gained by genome annotation, transcriptome sequencing, and the functional classification of single nucleotide polymorphisms to determine the roles of specific gene families. The *O. cumana* genome encodes 221 proteins with an *LRR* domain [102]. These genes are also annotated according to the presence of other domains (e.g., L domain, FBD domain, or F-box domain) and according to predicted molecular and cellular functions (e.g., ATP binding, cell wall organization, or oxidoreductase activity). Combined with the *S. gesnerioides* transcriptome analysis discussed above, showing that haustorium genes are also enriched for *LRR* domains [114], we can begin to see the outline of a process in which parasitic plants overcome host resistance by targeting components of signal transduction pathways activated by the *R* genes containing LRR domains to block defense response cascades directly or indirectly. Beside, *LRR* domains, a secretome analysis of *Striga hermonthica* revealed a large number of cysteine-rich small proteins associated with protease and cell wall modification activities was also involved in *S. hermonthica–*host plant interaction [117]. To counter the virulence of parasitic plants, hosts also detect and respond to molecular signals secreted by parasitic plant. For example, a surface receptor *Cuscuta Receptor 1* (*CuRe1*) was also identified in tomato plants that responded to *Cuscuta* spp. peptide factor and activates immune response and identify parasitic plants in a manner similar to perception of microbial pathogens [118].

Recently, a high-throughput silencing approach was developed to study *NLR* proteins in *Nicotiana benthamiana* in which 257 VIGS constructs based on tobacco rattle virus were used to target 386 of the 403 identified proteins, providing an efficient strategy to discover new immune receptors [119]. Agrobacterium-mediated transformation, together with transcriptome analysis of differentially expressed genes in *S. gesnerioides*, was used to dissect the involvement of resistance cascades in cowpea that is being attacked by this parasite [115]. Transcriptome assembly to identify genes in *Striga* and *Orobanche* was used to investigate the involvement of virulence proteins on a genome-wide scale [120,121,122]. 

#### 3.4.4. Genome and Transcriptome of Parasitic Plants

Genomics, transcriptomics, proteomics, bioinformatics, biochemistry, and cell biology have all played major roles in the identification and functional characterization of pathogen and host proteins involved in plant–pathogen interactions. One of the key challenges when applying such methods to parasitic plants is the need to extract pure nucleic acids or proteins from the parasite during infection, without contaminating host material. The distinction between host and parasite is complicated by the extensive mutual HGT and high substitution rates in parasitic plant genomes, and this also makes it more difficult to construct accurate phylogenetic trees [116,123,124]. New dating approaches have been applied to solve the problem of long branch lengths in gene tree analysis, allowing the absolute divergence time of parasitic plants to be determined more precisely [123]. For example, Kim et al. [125] developed a protocol to study the movement of parasitic mRNA into the host plant.

Thus far, 25 parasitic plant genomes have been sequenced and assembled, including nuclear, plastome, and mitochondriome sequences. Ten nuclear genome sequences have been reported, namely, the holoparasite *Cynomorium* [126], *Viscum scurruloideum* [4], *Viscum album* [4], *Castilleja paramensis* [126], *Cuscuta campestris* [127], *Cuscuta australis* [128], *Hydnora visseri* [129], *Aphyllon epigalium* [130], *Striga asisatica* [131], and *O. cumana* [95]. These resources could promote the analysis of candidate parasite-specific genes and provide evidence for host-to-parasite, parasite-to-host, and bidirectional [116,124,132,133,134]. For example, Illumina sequencing of the *Cynomorium* plastid and mitochondrial genomes revealed that the plastome contigs assembled into inverted repeat (IR) regions and a large single copy region. All genes involved in photosynthesis (*ndh*, *atp*, *pet*, *psa*, *psb*, and *rbcL*) have been lost as anticipated, but the predominant IR region contains genes encoding an ATP-dependent Clp protease proteolytic subunit (clpP) and metal-resistance protein (*YCF1*) [130]. Evidence of HGT was presented showing the transfer of host mitochondrial genes into the *Cynomorium* mitochondrial and nuclear genomes and the intracellular transfer of *Cynomorium* mitochondrial and plastid genes into the nuclear genome [135].

Transcriptome sequencing allows the functional analysis of parasitic plant genomes, and the Parasitic Plant Genome Project (Available online: http://ppgp.huck.psu.edu/) mainly focuses on the identification of genes related to haustorium initiation and development by applying comparative transcriptomics to multiple stages of parasite growth and development in three species of Orobanchaceae: *T. versicolor* (a facultative hemiparasite) (Figure 1a), *S. hermonthica* (an obligate hemiparasite), and *P. aegyptiaca* (an obligate holoparasite) [3,120]. A core set of “parasitism genes” was identified that are enriched for proteases, cell wall-modifying enzymes, and proteins secreted during haustorium development. Genes encoding transporters (cationic amino acid transporter, major facilitator family protein, NOD26-like intrinsic protein, and an oligopeptide transporter) and regulatory proteins (transcription factors and receptor protein kinases) are co-expressed during the parasitic stages and may be required for haustorium development and function [114]. *NLR* resistance genes are found in all three species and are significantly enriched in *T. versicolor* and *S. hermonthica*, suggesting an underlying important function that may facilitate the future analysis of race/virulence in parasitic plants. 

The expression levels of the parasitism genes differed between the hemiparasites and holoparasite at the haustorium stage. The genes encoding cell wall-modifying enzymes (cellulase, Pectate Lyases, glycosyl hydrolases, and pectin methylesterase) and peroxidases were strongly expressed in both hemiparasites (*T. versicolor* and *S. hermonthica*) but not in *P. aegyptiaca*, although expression increased at a later stage of the life cycle [114]. There were also differences in expression between the facultative parasite (*T. versicolor*) and the obligate parasites. In *T. versicolor*, the haustorium initiation genes were primarily Ca^2+^ATPases, including genes coding for proteins with functions such as Ca^2+^-binding activity, Ca^2+^-transporting ATPase activity, Ca^2+^ transmembrane transporter activity, and cation-transporting ATPase activity. In contrast, the *S. hermonthica* haustorium initiation genes were enriched for a distinct set of gene ontology terms, including nucleotide binding and ATP-dependent helicase activity, suggesting that facultative and obligate parasitic plants used different underlying processes for haustorium initiation [114].

Transcriptomic studies have also shown that, once connections between the parasite and host plant are established, the host–parasite relationship relies on the transfer of nutrients and solutes from host to parasite via multiple transporters, including amino acid and sugar transporters [114]. For example, the transcriptomic analysis of *S. hermonthica* infected leaves and flower buds during the parasitism of maize and sorghum hosts identified transporters (primarily carbohydrate and amino acid transporters) as the most common functional class of parasitism genes, followed by cell wall-modifying enzymes [136]. Similarly, the de novo assembly and characterization of the *S. gesnerioides* transcriptome during the pre-haustorium and haustorium stages of infection (Figure 1c) revealed the strong induction of genes encoding cell wall-modifying enzymes and transporters, including sugar transporters, amino acid transporters, ATP-binding cassette-type transporters, ammonium transporters, phosphate transporters, nitrate transporters, and potassium transporters [136].

All candidate haustorium genes are valuable resources for future functional and evolutionary studies, which will help to determine whether they are secreted by the parasite and whether they influence parasite–host interactions. For example, upregulated haustorium genes that encode subtilisin-like serine proteases [114] are similar to those acting as virulence factors in bacterial pathogens [137]. However, serine proteases are often involved in protein degradation and processing, the hypersensitive response, and signal transduction in nonparasitic plants, so their specific role in the parasitic life cycle has yet to be determined [138,139]. Indeed, numerous questions remain concerning the functional role of core parasitism genes identified by genomic and transcriptomic studies. The three species of Orobanchaceae considered by the Parasitic Plant Genome Project feature 84 orthologous groups with no BLAST (basic local alignment search tool) hits against annotated genes in nonparasitic species (178, 180, and 139 unique genes in *T. versicolor*, *S. hermonthica,* and *P. aegyptiaca*, respectively) although a small number match predicted protein sequences in nonparasitic plants but the functions are currently unknown (6, 18, and 13 sequences in *T. versicolor*, *S. hermonthica,* and *P. aegyptiaca*, respectively) [108]. Three focal transcripts of *S. hermonthica* generate no BLASTx hits at all [136]. Genes of unknown function are also found in the *Cynomorium plastome* [135]. 

## 4. Models of Interaction and Co-Evolution between Parasitic Plants and Their Hosts 

**Figure 5:**
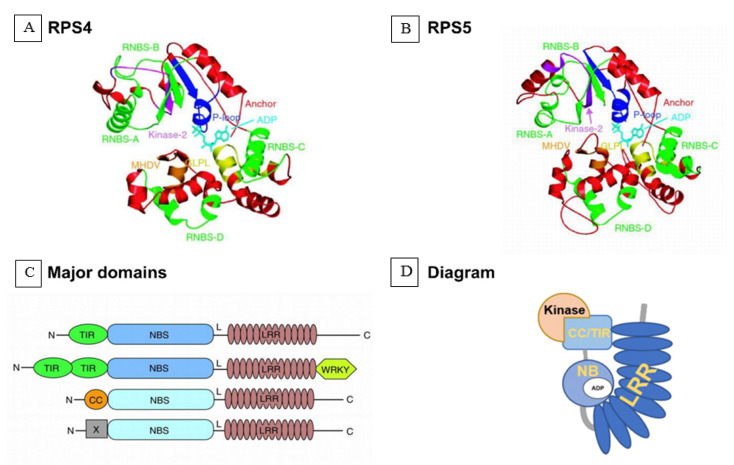
Predicted structures and models of nucleotide-binding site domain and a leucine-rich repeat domain (NB-LLR). (**A**) The structures of the NBS domains (nucleotide-binding site domain and a leucine-rich repeat domain) of TNL and *RPS4*. The protein structures are shown as ribbon diagrams and ADP (adenosine diphosphate) is shown as a stick model. TIR-type and CC-type *NBS* domains are made up of motifs: P-loop (or Walker A site, blue), *RNBS-A* (green), kinase-2 (or Walker B site, magenta), *RNBS-B* (green), *RNBS-C* (green), *GLPL* (yellow), *RNBS-D* (green), and *MHDV* (orange) [131]. Structural models for the NBS domain of *TNL RPS4* and CNL RPS5 of *Arabidopsis* were created by self-consistent mean-field homology modeling technique [132]. (**B**) The structures of the NBS domains of *CNL RPS5*; (**C**) major domains of NBL proteins. N, amino terminus; TIR, Toll/interleukin-1 receptor-like domain; CC, coiled-coil domain; X, domain without obvious CC motif; NBS, nucleotide binding site; L, linker; LRR, leucine-rich repeat domain; WRKY, zinc-finger transcription factor-related domain containing the WRKY sequence; C, carboxyl terminus; (**D**) Diagram of *NLR*.

*R* genes in plants play an important role in the recognition of pathogen virulence factors, which is required to induce resistance. They typically show dominant phenotypes, but recessive resistance genes have also been reported. As discussed above, most *R* gene products contain a nucleotide-binding ATPase domain and an *LRR* domain (Figure 5) and are, thus, described as *NLR* proteins [104]. The *LRR* domain includes individual repeats that recognize pathogen proteins [140]. Several *NLR* proteins have been described, including *MLA10, Sr50, RPP13, RPS4, RPS5*, *ZAR1,* and L6 [141]. This has led to the definition of two subclasses, namely, the Toll and interleukin-1 receptor subclass (TNL) [142] and the coiled coil subclass (CNL). Structural models of both have been constructed based on the Arabidopsis proteins RPS4 (ribosomal protein s4) (TNL) and RPS5 (CNL) using self-consistent mean-field homology modeling in the absence of ADP. This ligand was then added by inference from the *APAF-1–ADP* complex without further refinement of the models to illustrate the position of the nucleotide relative to the conserved motifs [143,144]. The Toll and interleukin-1 receptor, coiled coil, and LRR protein interaction domains have the ability to swap functions, such as the recognition and recruitment of transcription factors or other host proteins [104]. *RPS5* is normally activated when a second host protein (*PBS1*) is cleaved by the pathogen-secreted protease *AvrPphB*. The *AvrPphB* cleavage site within *PBS1* can be replaced with cleavage sites for other pathogen proteases, which then enables *RPS5* to be activated by these proteases, thereby conferring resistance against new pathogens [105].

### 4.1. Model of Defense Activated by Host NLR Proteins Triggered by the Parasitic Plant 

**Figure 6:**
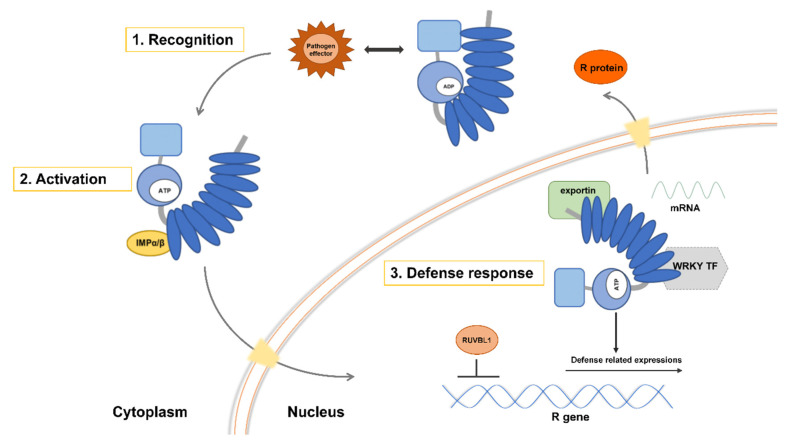
Putative model for defense activation by NLRs in host plant triggered by parasitic plant. Induction of defense responses by NB-LRRs (nucleotide-binding site domain and a leucine-rich repeat domain) proceeds in three stages. In some cases, kinase will take part in indirect recognition. In the first stage, the inactive *NLR* receptor (blue) perceives the presence of specific virulence proteins, called pathogen effector (brown), secreted from parasitic plant, then binds with pathogen effector. In some cases, kinase (also referred as host factor) will take part in indirect recognition. *NB-LRR* could indirectly recognize pathogen effector through *N*-terminal domain (CC or TIR) by an intermediary kinase. In the second stage, NB-LRR receptor is activated by a conformation change and ATP binding to NB domain. A highly conserved nucleotide-binding domain that is shared with apoptotic protease activating factor 1 (*APAF-1)*, various R-proteins, and *CED-4* (NB-ARC domain) is proposed to act as a molecular switch, cycling between ADP (repressed) and ATP (active) bound forms [132]. In the third stage, activated NB-LRR work in the nucleus to induce defense-related signaling and gene expression. *NLR* negative regulators of defense such as (*TIP49a*) transcription factor (TF) is inhibited. Alternatively, WRKY transcription factor (TF) may bind to *NLR* to positively regulate and induce defense expression. Chimeric proteins comprise domains typical for both intracellular type-R proteins (NBS–LRR proteins) and WRKY transcription factors [146], suggesting that WRKY TF binds to *NLR* closely. To cross the nuclear pore, *NLR*s with a classical nuclear localization signal will require importin-α and importin-β (light yellow) for import and export [104]. Last, specific defense-related mRNAs or proteins are exported through nuclear pore. *IMP*α/β: importin-α/β; R genes: *RPS5*.

The induction of defense responses by *NLR* proteins proceeds in three stages, as shown in Figure 6. The *LRR* region is an inhibitory domain, associating with the nucleotide-binding domain when there is no infection. The *N*-terminal coiled coil domain associates with a protein kinase such as PBS1 [104,105] (not shown in the model). In the first stage, the inactive *NLR* receptor (blue) perceives specific virulence proteins (pathogen effectors, shown in brown) secreted from the parasitic plant and binds to them (Figure 6, step 1). *NLR* proteins are highly specific, with each *NLR* protein capable of detecting only a limited number of effectors [105]. In some cases, the kinase (also described as a host factor) takes part in indirect recognition, enabling the *NLR* protein’s N-terminal domain to bind pathogen effectors via an intermediary kinase. In the second stage, the *NLR* receptor is activated by a conformation change and ATP binding to the nucleotide-binding domain, relieving the latter from LRR repression. The exchange of ADP for ATP at the nucleotide-binding domain may generate an activated, ATP-bound form of *NLR* [133]. Recently, a highly conserved nucleotide-binding domain shared byAPAF-1, various R proteins, and *CED-4* (the NB-ARC domain) was proposed to act as a molecular switch, cycling between ADP binding (repressed) and ATP binding (active) [145] (Figure 6, step 2). Finally, the activated *NLR* protein translocates to the nucleus to induce defense-related gene expression and corresponding signaling pathways (Figure 6, step 3). In the presence of the pathogen effector, the activated form of an *NLR* accumulates in the nucleus to initiate defense signaling.

Defense responses include a localized hypersensitive response that serves to prevent spread of infection by triggering cell death. *NLR* negative regulators of defense are inhibited during this response, including the transcription factor *RUVBL1* (*TIP49a*). Alternatively, WRKY transcription factors may bind to *NLR* to induce defense gene expression. *RUVBL1* (*TIP49a*) is a member of the AAA+ ATPase family (ATPases associated with various cellular activities), and *Arabidopsis TIP49a* (*RUVBL1*) can act as a negative regulator of some *R* gene functions [147]. WRKY transcription factors contain a highly conserved, ∼60 amino acid domain featuring the consensus sequence *WRKYGQK* and a zinc-finger motif [148,149]. WRKY transcription factors recognize the *cis*-regulatory element (T/A)TGAC(T/A), also known as the W-box, in the promoters of target genes [150,151]. Certain soybean (*Glycine max*) WRKY genes (*GmWRKY154*, *GmWRKY62*, *GmWRKY36*, *GmWRKY28,* and *GmWRKY5*) promote resistance to the soybean cyst nematode (*Heterodera glycines*) [152]. The presence of chimeric proteins featuring the domains of both intracellular R proteins (*NLR* proteins) and WRKY transcription factors suggests that these protein families work closely together [148]. To cross the nuclear pore, *NLR*s with a classical nuclear localization signal require importin-α and importin-β (light yellow) for import and export, respectively [104]. Finally, defense-related mRNAs or proteins are exported through the nuclear pore. 

### 4.2. Model of Antagonistic Host–Parasite Co-Evolution

**Figure 7:**
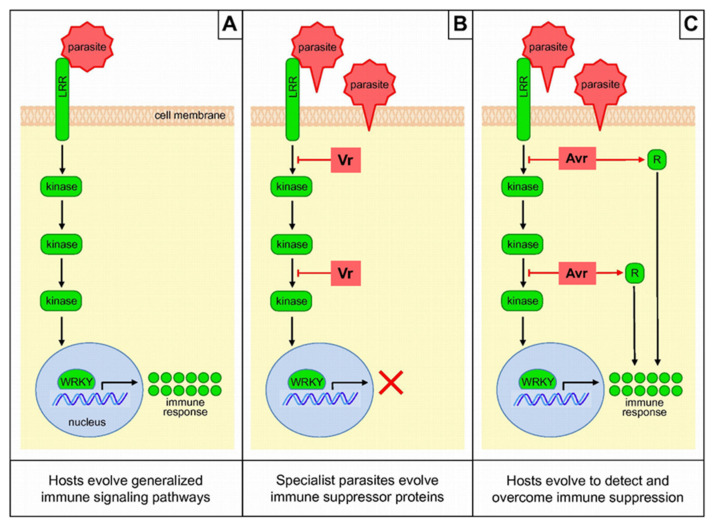
Parasitic plant-host antagonistic co-evolution. In figure (**A**), host plants evolve an antiparasite immune response that protects them from most parasitic plant invasion. New parasitic plant race evolves suppressive virulence mechanisms in figure (**B**), selecting the plant hosts to counter-evolve secondary immune mechanisms in figure (**C**). Figures (**B**) and (**C**) can then repeatedly cycle in an evolutionary “arms race”. Figures are adapted from Keebaugh et al. [151].Vr = virulence, Avr = avirulence, *LRR =* leucine-rich repeat, WRKY = Zinc-finger transcription factor-related domain containing the WRKY sequence.

A remarkable consensus has emerged concerning the genetic basis of virulence and resistance in typical interactions between plants and microbial parasites, and we can build a similar hypothesis for the interaction with parasitic plants based on a co-evolution model (Figure 7). When host plant R proteins win a “match” against the race-specific effectors or virulence proteins of parasitic plants, then the effectors become redefined as avirulence (Avr) proteins. The nature of R–Avr interactions is now well understood [151]. Host plants have receptor proteins (including the *NLR* proteins discussed above) that perceive parasite proteins and trigger responses that confer immunity [104,119] via the activation of WRKY transcription factors [151]. To circumvent host immunity, parasitic plants evolve new virulence proteins that disrupt the host defense pathways. To overcome these virulence effectors, host plants adapt their R proteins to recognize the new virulence proteins [152,153,154], enabling the reactivation of the downstream response, and so the cycle continues [155].

### 4.3. Drivers of Pathogen Effector Evolution

**Figure 8:**
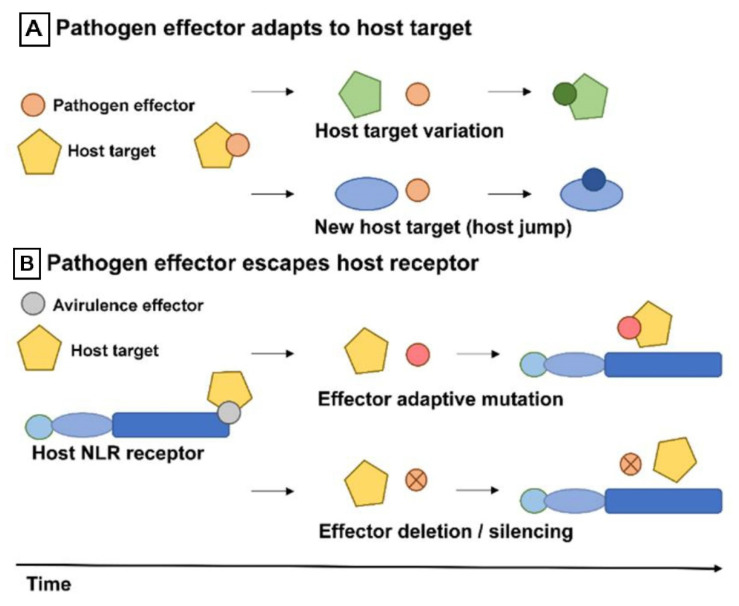
Forces of virulence effector evolution of parasitic plant. (**A**) Parasitic plant virulence effectors adapt to new host targets. Natural variation in a host targets or changes in the biotic environment, for example, a host jump, drive effector adaptation. This results in effectors binding or acting on a variant of the original target or on a totally new host target. (**B**) Effectors evade host immune receptors. Effectors also evolve to evade recognition by host immune receptors, for example, *NLR*. This can occur through adaptive mutations that result in stealthy effectors, which avoid host recognition but retain virulence activity. Alternatively, effector genes can also escape host immunity through pseudogenization, deletion, or gene silencing. Figure adapted from Upson et al. [156].

The evolution of pathogen effectors is driven by two forms of selection pressure, adaption to targets in the host and adaption to evade detection by the host (Figure 8). The interplay between these dynamic selection pressures creates an inherently unstable biotic environment for pathogen effectors, accelerating effector evolution [156]. One perfect case to study what drives the evolution of virulence effectors in parasitic plant is natural populations of parasitic plant parasitizing different host species based on gene expression level. A number of differential *S. hermonthica* transcripts were identified depending on whether it grew on maize or sorghum [136]. These differential transcripts including genes are involved in defense mechanisms and pathogenesis, some of which might be parasite effectors that subdue host defense [4]. Pathogen effectors show marked patterns of gene evolution following host jumps, where there is extreme pressure to adapt to new host targets (Figure 8a). Parasitic plants also suppress plant host defense by producing a battery of molecules (effectors), just like bacterial and fungal pathogens [157,158]. Host specialization that leads to evolutionary divergence depends on reciprocal single amino acid changes that tailor the pathogen effector to a specific host protein that is disabled. Thus, small changes in either the host or the pathogen can allow pathogens to jump to another host species [159]. For example, orthologous protease inhibitors from the oomycetes *Phytophthora infestans* and *Phytophthora mirabilis* have adapted to target unique proteases in different hosts, allowing the pathogens to specifically target potato (*Solanum tuberosum*) and the four o’clock flower (*Mirabilis jalapa*), respectively [159].

Avirulence effectors that are detected by plant receptors are prominent examples of rapid evolutionary adaptations. Notably, parasite pathogen effector variants with an excess of nonsynonymous polymorphisms (amino acid replacements) that escape detection by the host plant while retaining virulence (Figure 8b) can carry extreme signatures of adaptive evolution [160]. In a recent case, three avirulence effectors from the rice blast fungus *Magnaporthe oryzae* (*AVR-Pik*, *AVR-Pia*, and *AVR-Pii*), matching three rice resistance genes (*Pik*, *Pia*, and *Pii*), have been validated by comparative genomics. Among these effectors, *AVR-Pik* (in which allelic variants only carry nonsynonymous polymorphisms) binds to an interface of the *NLR* receptor *Pik-1* [161,162]. Effector genes can evolve through a birth and death process via chromosomal rearrangements, resulting in significant levels of presence/absence of polymorphisms within pathogen populations (Figure 8b) [163]. Effector genes can escape host recognition through pseudogenization [164], deletion [165], or gene silencing [166]. Many effector gene loci segregate as presence/absence polymorphisms, leading to a mosaic of effector genes within species such as *Magnaporthe oryzae* [156] and the cereal pathogen *Zymoseptoria tritici* [167].

Interestingly, the analysis of pathogen genomes has revealed that effector genes often arise in highly repetitive but gene-sparse regions rich in transposable elements [164,166,168]. This genome architecture has affected nearly every aspect of effector evolution, including transcriptional control, mutation rates, loss of function, and deletions [151]. Interestingly, the recent availability of parasitic plant genome sequences has shown that these, too, are rich in transposable elements, suggesting that effector genes may evolve in a similar manner [4,113,133].

This arms race probably stands on the foundation of gene-for-gene interaction between plant resistance and pathogen avirulence factors [141]. In theoretical models, the frequencies of resistance and virulence alleles in a population progress in an infinite cycle, sometimes called the boom-and-bust cycle, because of the frequent dramatic rise and fall in the effectiveness of resistance genes against pathogen populations in agriculture. Researchers also offer mathematical models for the co-evolution of host and parasites in terms of genetic diversity to test the gene-for-gene model, as discussed in a comprehensive review [169]. 

## 5. The Effect on Parasitic Plants

Clustered regularly interspaced short palindromic repeats/CRISPR associated protein 9 (CRISPR/Cas9)-mediated mutagenesis and RNA interference (RNAi) silencing have both been used to disrupt SL biosynthesis in host plants, aiming to suppress the germination and, thereby, infection of parasitic weeds. CRISPR/Cas9 is a form of adaptive immunity found in bacteria and archaea, which has been engineered as a powerful gene editing tool that has been applied in more than 20 crop species [170]. It has been applied in rice to disrupt the *CCD7* gene, reducing SL biosynthesis and inhibiting the germination of *S. hermonthica* [171] and similarly in tomato (*Solanum lycopersicum*) to disrupt the *CCD8* gene, inhibiting the germination of *P. aegyptiaca* [172]. In both cases, resistance was transmitted as a transgene-free trait by segregating the Cas9 cassette from the induced mutation. The tomato *CCD8* gene has also been targeted by RNA interference (RNAi), in which the expression of double-stranded RNA corresponding to the target gene causes post-transcriptional silencing [173]. Interestingly, targeting the tomato *CCD8* gene by RNAi led to the faster development of parasite tubercles when pregerminated *O. ramosa* seeds were used to infect the plant, suggesting that SLs inhibit parasite development after attachment [173]. It is possible that auxin levels or transport efficiency increased as a result of the decreased *CCD8* expression in the RNAi lines, as previously reported for the *Arabidopsis* SL-deficient mutant *max4* [174]. 

Eukaryotes have evolved several gene-silencing pathways to defend against viruses, mediated by small interfering RNA (siRNA) molecules 21–24 nt in length [175,176,177]. Although the natural purpose of these pathways is to recognize and attack of viral nucleic acids invading the cell, the components can be harnessed to recognize specific mRNA molecules, thus suppressing gene expression by either destroying the mRNA or blocking protein synthesis [176,178]. This has been exploited to develop host-induced gene silencing (HIGS) technology to control plant pathogens, in which the plant expresses siRNAs targeting gene expression in the attacking parasite [179]. RNAi or HIGS strategies have been used to try to affect gene expression in parasitic plants such as *Triphysaria versicolor* [180,181], *Cuscuta pentagona* [182], *S. hermonthica* [183], *S. asiatica* [184], *O. aegyptiaca* [181,185,186], and *Phelipanche ramosa* [173,187]. A β-glucuronidase (GUS) silencing signal can move from the transgenic host to another host and there silence GUS through the parasite *Triphysaria versicolor* as a physiological bridge [180]. Interspecific silencing of a SHOOT MERISTEMLESS-like (*STM*) gene in dodder driven by a vascular promoter in transgenic host plants disrupts dodder growth, demonstrating the efficacy of interspecific small RNA-mediated silencing of parasite genes [182]. Three *O. aegyptiaca* genes were suppressed to induce parasite mortality by virus-induced gene silencing (VIGS) and hairpin silencing on host tomato [186], which impaired expression of essential parasite virulent genes.

### The Effect on Host Plants 

Although the principal effect of suppressing the SL biosynthesis pathway is to inhibit the parasite, SLs are also required for normal plant development by inhibiting shoot branching/tillering and regulating the growth of primary and lateral roots [63,64,188]. Thus, the rice *ccd7* mutants discussed above exhibited stunting and a striking increase in tillering [171,189,190]. Similarly, tomato *ccd8* mutants were stunted, with increased shoot branching and adventitious root growth, and similar phenotypes were observed in *Arabidopsis*, tobacco (*Nicotiana tabacum*), and kiwifruit (*Actinidia deliciosa*) [170,191,192]. In wild-type tomato plants, *P. ramosa* infection reduces the root biomass, whereas both root and shoot biomass were affected when the same parasite infected tomato plants expressing the *CCD8* RNAi construct [173]. One way to avoid a dwarf phenotype is by grafting. Genome editing could be applied to rootstock already resistant to fungal pathogens, viruses, and nematodes, and this could be grafted to a wild-type scion in order to simultaneously achieve parasite resistance and normal growth [172]. Another solution is the application of the synthetic SL analog GR24, which can reduce the tiller number to wild-type levels in rice *ccd7* and *ccd8* mutants [171,189]. 

For farmers, the benefits of mutant lines with increased parasite resistance must be balanced against any trade-off against crop quality and yield. Mutation of the *CCD8* gene in tobacco caused a loss of shoot biomass [191] but mutating the same gene in tomato resulted in the production of numerous additional fruits, although these were smaller than wild-type fruits [174]. The depletion of SL in root exudates not only inhibits the germination of parasitic plants but also impairs symbiotic relationships with arbuscular mycorrhizal fungi [71,193]. This is because *CCD7* not only regulates branching, but also arbuscular mycorrhizal symbiosis [194]. More research is required to determine the possibilities to manipulate SLs in order to suppress the growth of parasitic plants while retaining normal growth characteristics and beneficial relationships with symbionts. 

## 6. Conclusions 

The intensification of agriculture has led to a surge in the prevalence and dispersal of parasitic plants that utilize crop species as their hosts, resulting in extensive yield losses. Although resistant crop varieties can be engineered or bred, some parasitic plants rapidly evolve as new races to break the resistance and establish infestations. Therefore, it is essential to understand the virulence mechanisms of parasitic plants and the corresponding host plant defensive responses. Phenotypic quantification of the infection level is the primary approach to identify the race or pathotype at the shoot emergence stage. Host plants deploy multiple layers of defenses including physical barriers (the cuticle and cell wall) and constitutively produced metabolites, but one of the major strategies to avoid parasitism is reducing the exudation of germination stimulants, particularly SLs released by the roots. We illustrated the history of parasitic plant race evolution and host resistance using *O. cumana* vs. sunflower and *S. gesnerioides* vs. cowpea as model systems. Genetic, genomic, and transcriptomic studies have shown that the major host resistance components are R genes encoding *NLR* domain proteins that play an important role in host immunity by recognizing parasite virulence factors. The induction of host defense responses by *NLR* proteins’ model is proposed in three stages: recognition, activation, and defense response. We also proposed a hypothesis for the virulence effector evolution of parasitic plants based on genetic basis of typical interactions between plants and microbial parasites. The evolution of pathogen effectors is driven by two forms of selection pressure: adaption to targets in the host and adaption to evade detection by the host. Transcriptomic and genomic studies are also beginning to identify the virulence effectors and corresponding signaling pathways in parasitic plants, building into a rich information resource for future studies. Biotechnology-based approaches (CRISPR/Cas9 and RNAi) has resulted in reduced host infection by parasitic plants, but it is important to ensure that the endogenous functions of SLs are not disrupted, as well as preserving the crosstalk with other hormone pathways. In the future, gene co-expression network analysis could be used to select parasite gene candidates for targeted knockdown to develop parasitic weed-resistant crop varieties.

## Abbreviations

HIGS	Host-induced gene silencing
HGT	Horizontal gene transfer
LRR	Leucine-rich repeat
NLR	Nucleotide-binding and leucine-rich repeat domain
PR	Pathogen-related protein gene
QTL	Quantitative trait loci
R	Resistance gene
SiRNA	Small interfering RNA
SL	Strigolactone
HGT	Horizontal gene transfer
GR24	Synthetic analog of strigolactones
WRKY	Zinc-finger transcription factor-related domain containing the WRKY sequence
TF	Transcription factor
Avr	Avirulence
Vr	Virulence
RNAi	RNA interference
Crispr/Cas9	Clustered regularly interspaced short palindromic repeats/CRISPR associated protein 9
